# Lead-Free Halide Perovskite Nanocrystals for Light-Emitting Diodes

**DOI:** 10.3390/ma16186317

**Published:** 2023-09-20

**Authors:** Do-Young Kim, Jae-Geun Jung, Ye-Ji Lee, Min-Ho Park

**Affiliations:** 1 Department of Materials Science and Engineering, Soongsil University, 369 Sangdo-ro, Dongjak-gu, Seoul 06978, Republic of Korea; kdy2269@gmail.com (D.-Y.K.); qwert59211@gmail.com (J.-G.J.); leeyejey@gmail.com (Y.-J.L.); 2 Department of Green Chemistry and Materials Engineering, Soongsil University, 369 Sangdo-ro, Dongjak-gu, Seoul 06978, Republic of Korea; 3 Integrative Institute of Basic Science, Soongsil University, 369 Sangdo-ro, Dongjak-gu, Seoul 06978, Republic of Korea

**Keywords:** halide perovskite light-emitting diodes, lead-free halide perovskite nanocrystals, perovskite nanocrystal emitters, eco-friendly perovskite nanocrystals

## Abstract

Lead-based halide perovskite nanocrystals (PeNCs) have demonstrated remarkable potential for use in light-emitting diodes (LEDs). This is because of their high photoluminescence quantum yield, defect tolerance, tunable emission wavelength, color purity, and high device efficiency. However, the environmental toxicity of Pb has impeded their commercial viability owing to the restriction of hazardous substances directive. Therefore, Pb-free PeNCs have emerged as a promising solution for the development of eco-friendly LEDs. This review article presents a detailed analysis of the various compositions of Pb-free PeNCs, including tin-, bismuth-, antimony-, and copper-based perovskites and double perovskites, focusing on their stability, optoelectronic properties, and device performance in LEDs. Furthermore, we address the challenges encountered in using Pb-free PeNC-LEDs and discuss the prospects and potential of these Pb-free PeNCs as sustainable alternatives to lead-based PeLEDs. In this review, we aim to shed light on the current state of Pb-free PeNC LEDs and highlight their significance in driving the development of eco-friendly LED technologies.

## 1. Introduction

In recent years, metal halide perovskites (MHPs) have received considerable attention as highly promising materials for a range of optoelectronic applications, including solar cells, light-emitting diodes (LEDs), photodetectors, lasers, and photocatalysts [1,2,3,4,5,6,7,8,9,10,11,12]. Notably, lead (Pb)-based halide perovskites (LHPs) have emerged as particularly appealing. This is due to their exceptional optoelectronic properties, which are characterized by high photoluminescence quantum yield (PLQY), long carrier diffusion length, high charge carrier mobility, high defect tolerance, and narrow emission spectra [13,14,15,16,17,18,19,20]. These properties make these materials highly promising candidates for next-generation LEDs. Moreover, a reduction in the crystal size toward the nanometer scale has a substantial impact on the material properties, thereby enabling deliberate adjustment of the optoelectronic characteristics [21,22,23,24,25]. Applications of MHP nanocrystals (PeNCs) have already been established in various fields [26,27,28,29,30,31,32], and their device applications are expected to be commercialized.

However, the presence of Pb^2+^ in LHPs poses significant challenges to their widespread commercialization. Pb^2+^ is highly soluble in water, which introduces potential risks to the environment and human health throughout the product life cycle, including disposal. Exposure to Pb can result in various adverse health effects, including neurological disorders, gastrointestinal issues, insomnia, coma, and convulsions. The inherent toxicity of Pb raises concerns regarding its environmental impact and human safety, making LHPs incompatible with the restriction of hazardous substances (RoHS) directive and industrial reliability standards [33,34,35]. Therefore, there has been growing interest, and various research efforts have focused on exploring and developing Pb-free PeNCs as promising alternatives to address these intrinsic challenges.

The development of Pb-free perovskites is a crucial alternative strategy for the commercialization of MHP-based devices by substituting Pb cations with nontoxic metal cations, such as tin (Sn), bismuth (Bi), antimony (Sb), or transition metals such as copper (Cu). To achieve this goal, researchers have aimed to eliminate toxicity concerns associated with Pb while achieving comparable or enhanced optoelectronic performance [36,37,38,39,40].

In this review, we provide a comprehensive overview of recent advances in Pb-free PeNCs for LED (PeNC-LED) applications. We discuss the synthetic strategies employed for the development of Pb-free PeNCs, including the selection of nontoxic metal cations and the optimization of crystal structures. Additionally, we examine the optoelectronic properties of Pb-free PeNCs, such as their photoluminescence (PL) characteristics, band structure, and emission mechanism. Moreover, we explore the strategies employed to enhance the stability and operational lifetime of Pb-free PeNC-LEDs and discuss the challenges by addressing key research directions and prospects for the development of Pb-free PeNC-LEDs.

## 2. B-Site Metal Candidates for Pb-Free Perovskite Nanocrystals

**Figure 1:**
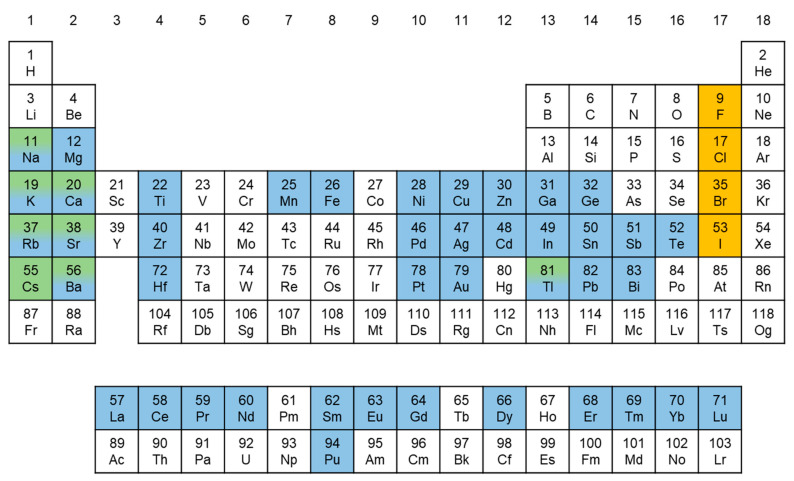
Periodic table of the elements that have the potential to constitute perovskite compositions (A-site: green; B-site: sky-blue; and X-site: orange).

In the context of traditional LHPs, the general chemical formula can be expressed as ABX_3_, wherein A represents an inorganic monocation such as Cs^+^, Rb^+^, or K^+^ and an organic monocation such as the methylammonium cation (MA^+^) and the formamidinium cation; B denotes a bivalent Pb^2+^ ion; and X represents halide ions such as Cl^–^, Br^–^, or I^–^ [41,42]. The stability of three-dimensional (3D) ABX_3_ perovskite structures can be evaluated using Goldschmidt’s tolerance factor *t* (1) and the octahedral factor *μ* (2):

(1) $$t=\frac{r_{A}+r_{X}}{\sqrt{2}(r_{B}+r_{X})}$$

(2) $$\mu =\frac{r_{B}}{r_{X}}$$

where *r_A_*, *r_B_*, and *r_X_* correspond to the ionic radii *A*, *B*, and *X*, respectively. Stable perovskite crystal structures are formed when these two factors are in the ranges of 0.8 ≤ *t* ≤ 1.0 and 0.44 ≤ *μ* ≤ 0.90 [41,43,44,45]. However, as the ionic radius of the A-site cation increases, which leads to a larger tolerance factor, lower-dimensional perovskite structures are formed [46,47,48]. Such dimensions and crystal structure variations result in MHPs having diverse band structures and photophysical and optoelectronic properties, making them promising candidates for various applications. Figure 1 shows the elements in the periodic table denoted by distinct colors (A-site: green, B-site: sky-blue, and X-site: orange), considered potentially viable or having been employed at the *A*, *B*, and *X* positions of the MHPs [49,50,51,52,53,54]. Extensive research is being conducted on MHPs with various compositions to progressively enhance their performance across diverse fields.

Furthermore, MHPs based on toxic metals, such as Pb and Cd, encounter challenges for commercialization owing to the RoHS directive. Thus, research on Pb-free MHPs is essential for their industrial applications. To substitute non-toxic metals for toxic Pb in LHPs, attention should be paid to the ionic radius and charge neutrality of the substituting metal elements. The selection of elements must satisfy the requirements of an appropriate ionic radius to maintain the structural integrity of the perovskite lattice. Moreover, substitutions should ensure charge neutrality within the crystal structure to maintain overall electrostatic balance [55]. By carefully considering these factors, it is possible to identify suitable elements for effective Pb substitution in LHPs. This leads to the development of Pb-free MHPs with the desired properties for optoelectronic device applications, including LEDs. Among the promising elemental candidates, we focus on Sn-, Bi-, Sb-, Cu-based, and double PeNCs and provide an overview of their applications in PeNC-LEDs.

### 2.1. Sn-Based Perovskite Nanocrystals

**Figure 2:**
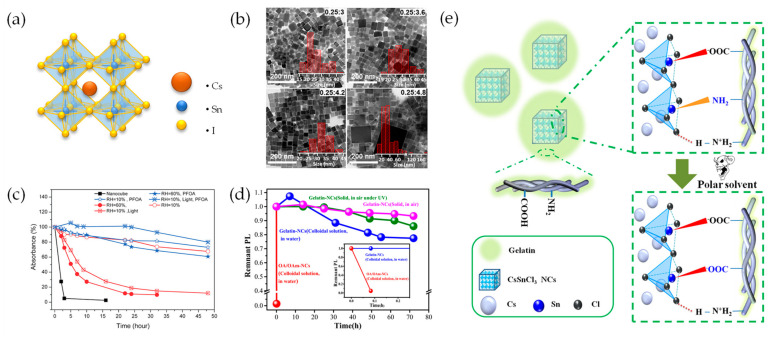
(**a**) Crystal structure of CsSnI_3_; (**b**) TEM images of CsSnI_3_ PeNCs with different ratios of Cs:Sn. Insets indicate histograms of the average particle size of each CsSnI_3_ NC; (**b**) Reprinted with permission from Ref. [58]. Copyright, 2021 American Chemical Society. (**c**) The variation of absorbance over time intensity at 620 nm under various conditions. The temperature and light intensity were 25 °C and 1200 mW/cm^2^ for all measurements; (**c**) Reprinted with permission from Ref. [59]. Copyright, 2017 American Chemical Society. (**d**) Remnant PL values of gelatin-NCs and oleic acid (OA)/oleylamine (OAm)-NCs over time under exposure to air, UV irradiation, and water. (Inset: remnant PL of gelatin-NCs and OA/OAm-NCs in 0.1 h). (**e**) Schematic image of gelatin coordinate with Sn in CsSnI_3_ NCs; (**d**,**e**) Reprinted with permission from Ref. [61]. Copyright 2020, Elsevier.

Sn belongs to group 14 of the periodic table, which is the same group as Pb, and possesses an equivalent valence configuration, resulting in the ability to retain electrical neutrality within the ABX_3_ crystal structure (Figure 2a). Moreover, Sn cations have an ideal cubic structure surrounded by an octahedron of anions. These characteristics have led to extensive investigations into Sn as a promising B-site metal replacement for Pb. However, ABX_3_-structured Sn-based perovskites have been observed to spontaneously undergo rapid decolorization and conversion to the A_2_BX_6_ formula after exposure to atmospheric conditions, implying a rapid degradation phenomenon [56]. Such structural changes adversely affect the chemical and luminescence properties of Sn-based PeNCs (Sn-PeNCs) [57]. Therefore, significant efforts have been made in recent years to investigate the structural and luminescent properties of Sn-PeNCs.

Precise control of the precursor ratios over various ranges is crucial for achieving a low density of structural defects within the lattice of the CsSnX_3_ NCs [58]. This is because the defect formation energy is intricately linked to the chemical potential of each constituent involved in the reaction. Figure 2b shows bright-field transmission electron microscopy (TEM) images of CsSnI_3_ NCs with different Cs:Sn ratios. As the Sn content increases, the average size of the cubic CsSnI_3_ NCs also increases. For instance, an average size of 26 nm was obtained at a Cs:Sn ratio of 0.25:3 and 37 nm at a ratio of 0.25:4.8. Additionally, byproducts, which are presumed to be unreacted species or reaction intermediates, were observed in CsSnX_3_ NCs with a ratio of 0.25:4.8. Furthermore, at the higher Sn ratio of 4.8, large crystals exceeding 200 nm were formed, larger than those at ratios of 3, 3.6, and 4.2. These results indicate that the precursor ratio plays a crucial role in determining the size distribution and purity of CsSnI_3_ NCs during synthesis.

By consistently addressing the challenge of preventing the oxidation of Sn(II) to Sn(IV), the performance of Sn-based MHPs has demonstrated consistent improvement. The low stability of CsSnBr_3_ NCs can be enhanced by the precise synthesis of CsSnBr_3_ cubic nanocages, which uses a simple hot-injection colloidal approach [59]. The fabrication of the nanocages can be carefully regulated by selecting suitable precursors, such as MgBr, and adjusting the reaction temperature, resulting in their formation through a self-assembly-driven process. MgBr was used as the bromide source because Mg^2+^ has a smaller ionic radius, which, according to tolerance factor theory, prevents its penetration into the perovskite structure. Uncoordinated 1-octadecene (ODE) was chosen as the solvent, and oleic acid (OA) and oleylamine (OAm) were selected as ligands. During this process, injection of the Cs-oleate precursor into the Sn-Br precursor solution resulted in a rapid color change, turning the solution dark red within a few seconds. This color transformation indicates the successful formation of CsSnBr_3_ nanocages.

A surface treatment with perfluorooctanoic acid (PFOA) also substantially enhanced the stability of CsSnBr_3_ cubic nanocages [59]. PFOA exhibits a stronger electron-withdrawing capability than Br^−^, which passivates the surface of the CsSnBr_3_ nanocages. It forms a stronger interaction with Sn^2+^, effectively hindering oxidation. Additionally, PFOA induces steric hindrance, further enhancing the stability of the CsSnBr_3_ nanocages. As shown in Figure 2c, the pristine CsSnBr_3_ nanocage films show gradual disintegration, with approximately 30% decomposition after 48 h under various desiccation conditions, whereas the PFOA-functionalized CsSnBr_3_ nanocage film exhibits a relatively slow decomposition rate of 25% in a moisture-free environment. When the films were exposed to illumination, the pristine CsSnBr_3_ films degraded rapidly, with over 95% decomposition within 24 h. Conversely, the PFOA-functionalized CsSnBr_3_ film exhibited a slight degradation of approximately 10% under the same conditions. Therefore, water is a critical factor that directly influences the stability of CsSnBr_3_ nanocages, and exposure to light accelerates degradation by the decomposition process.

Surface passivation using polymers is another effective strategy for enhancing the stability of Sn-PeNCs [60,61,62]. The incorporation of gelatin, an amphoteric polymer obtained as a partial product of collagen hydrolysis, is crucial for providing resistance against water degradation [61]. The hygroscopic nature of the gelatin network effectively stabilizes and protects CsSnCl_3_ NCs from water-induced damage. Moreover, the high molecular weight of gelatin allows tight coating and isolation of the PeNCs, effectively suppressing self-aggregation and oxidation.

Accordingly, gelatin-coated CsSnI_3_ nanocrystals (gel-CsSnI_3_ NCs) have been shown to exhibit notably enhanced stability under harsh environmental conditions when compared to pristine OA/OAm-based CsSnI_3_ NCs (Figure 2d). The PL intensity of the gel-CsSnI_3_ NCs was maintained above 93.28% and 85% for 72 h under dark conditions and UV irradiation, respectively (pink and green lines). By contrast, the water resistance test revealed that the PL stability of pristine CsSnI_3_ NCs degraded by over 95% within 5 min. The low stability of pristine CsSnI_3_ NCs could originate from the addition of water to the PeNC solution in cyclohexane, resulting in delamination, ligand loss, self-aggregation, PL quenching, and even decomposition. On the contrary, the PL intensity of gel-CsSnI_3_ NCs exhibited minimal change after 5 min (Figure 2d, inset) and remained above 75% after 72 h (blue line).

Based on these results, Figure 2e presents a schematic of the proposed interaction mechanism between the gelatin and CsSnCl_3_ NCs. The interaction between gelatin and the CsSnCl_3_ NCs involves the coordination of the carboxylate and amino groups of gelatin with the Sn atoms in the NCs. Additionally, the protonated amino groups form hydrogen bonds with the Cl atoms. The numerous functional groups in gelatin lead to the occupation of active sites on the NC surface, creating a “rich ligand” state. As a result, comprehensive passivation of the NC surface by gelatin effectively inhibits the formation of surface defects. Therefore, the surface passivation strategy enhances the stability of Sn-PeNCs under atmospheric conditions, highlighting their potential for various applications.

Sn-PeNCs have been extensively studied as environmentally friendly Pb-free PeNCs due to their promising characteristics, including the same ns^2^ valence electron configuration and similar ion radii (Sn:1.35 Å, Pb:1.49 Å). They enable the substitution of Pb cations within the ABX_3_ structure while maintaining charge neutrality [63]. To enhance their structural stability, numerous studies have been conducted on controlling precursor ratios and surface engineering through the introduction of additives such as perfluoroalkyl acids and polymers. However, their practical applications have been limited owing to a lack of research on suitable additives for preventing Sn oxidation. Consequently, current research efforts have focused on enhancing the stability of Sn-PeNCs by exploring additives that can effectively inhibit Sn oxidation.

### 2.2. Bi-Based Perovskite Nanocrystals

The electronic properties of the LHPs can be attributed to the electronic configuration of Pb^2+^, which is characterized as 6*s*^2^6*p*^0^. This configuration enables the hybridization of the Pb 6*s* orbital with the halogen *p* orbital for valence band formation and of the Pb 6*p* orbitals with halogen *p* orbitals for conduction bands. This leads to the observed electronic properties. The presence of shallow defect states in LHPs is closely linked to the strong antibonding interactions that occur between the Pb 6*s* and halide 5*p* orbitals within the valence band [64,65].

Bi, which belongs to the same period as Pb, possesses a cationic electron configuration similar to that of Pb^2+^, featuring a [Xe] 4*f*^14^5*d*^10^6*s*^2^ electron arrangement. The presence of a 6*s*^2^ lone pair is a distinctive characteristic of Bi that enables it to displace Pb by a heterogeneous substitution mechanism, resulting in the replacement of Pb^2+^ with Bi^3+^ [66]. Moreover, Bi is an eco-friendly alternative to Pb, offering low toxicity and high stability [67,68,69,70].

**Figure 3:**
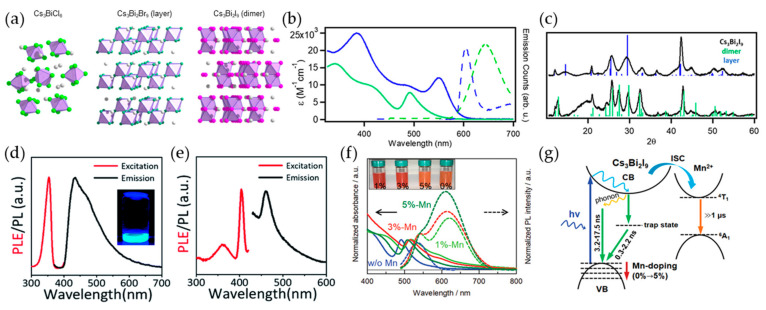
(**a**) Crystal structures of Cs_3_BiCl_6_, Cs_3_Bi_2_Br_9_, and Cs_3_Bi_2_I_9_ in bulk; (**b**) extinction coefficient (solid lines) and PL (dashed lines) spectra for Cs_3_Bi_2_I_9_ polymorphs (blue: layered, green: dimer); (**c**) Powder X-ray diffraction (XRD) patterns for Cs_3_Bi_2_I_9_ polymorphs; (**a**–**c**) reprinted with permission from Ref. [75]. Copyright 2019, American Chemical Society. (**d**) PL excitation (PLE) and PL spectrum of Cs_3_BiBr_6_ NCs. PL at an excitation wavelength of 360 nm and PLE at an emission wavelength of 440 nm; (**e**) PL and PLE spectra of Cs_3_Bi_2_Br_9_ NCs. PL under an excitation wavelength of 358 nm and PLE at an emission wavelength of 461 nm; (**d**,**e**) reprinted with permission from Ref. [87]. Copyright 2020, Royal Society of Chemistry. (**f**) Absorption (solid lines) and PL (dashed lines) spectra of different levels of Mn-doped Cs_3_Bi_2_I_9_ NCs, respectively. Inset: the appearance of different Mn-doped Cs_3_Bi_2_I_9_ NCs; (**g**) emission mechanisms of Cs_3_Bi_2_I_9_ with Mn^2+^ doping; (**f**,**g**) reprinted with permission from Ref. [88]. Copyright 2021, Wiley-VCH.

When Bi replaces Pb in LHPs, maintaining electrical neutrality requires the substitution of three Pb^2+^ ions with two Bi^3+^ ions, leading to the formation of A_3_B_2_X_9_ structures within the ABX_3_ framework. Leng et al. first reported the synthesis of MA_3_Bi_2_Br_9_ NCs that exhibited an emission wavelength of 430 nm and a maximum PLQY of 12% [71]. Subsequently, numerous studies have been conducted on the synthesis of Bi-PeNCs [69,70,71,72,73,74]. However, the inconsistent optical properties observed for various colloidal Bi-PeNC synthesis methods indicate the presence of various crystal structures. The combination of Cs^+^ and Bi^3+^ cations with halide anions can generate Cs_3_BiX_6_ and Cs_3_Bi_2_X_9_ (Figure 3a) [75]. Cs_3_Bi_2_Br_9_ possesses a two-dimensional (2D) layered structure in the P$\bar{3}$m1 space group, and one-third of its B-site cation positions are replaced by vacancies. By contrast, dimer structures such as Cs_3_Bi_2_I_9_ are formed by Cs^+^ or MA^+^ cations in the face-sharing octahedra and belong to the P6_3_/mmc space group. The bandgap calculated by density functional theory (DFT) using the Vienna ab initio simulation package indicates that the layered structure has a smaller bandgap than the dimer structure, which can be attributed to its smaller lattice constant and volume [76,77]. By changing the A-site in Cs_3_Bi_2_I_9_ from Cs^+^ to Rb^+^ or K^+^, the transformation from a dimer to a layered structure can be observed, leading to a transition from an indirect bandgap to a direct bandgap. However, when Rb^+^ or K^+^ cations are used at the A-site of the A_3_Bi_2_I_9_ structure, the smaller size of these ions results in a lower-symmetry distorted lattice with the P2_1_/n space group [78].

To investigate the bandgap characteristics associated with the Cs_3_Bi_2_I_9_ crystal structure, colloidal Cs_3_BiX_6_ and Cs_3_Bi_2_X_9_ NCs were synthesized using the hot-injection method and anion exchange with trimethylsilyl halides (TMS-X) [75]. The direct synthesis of Cs_3_Bi_2_I_9_ leads to the formation of a dimer rather than a layered structure [79,80,81]. Because the direct synthesis of layered Cs_3_Bi_2_I_9_ is challenging, layered Cs_3_Bi_2_I_9_ can be achieved through anion exchange with TMS-I. When comparing the optical properties of dimeric Cs_3_Bi_2_I_9_ with those of layered Cs_3_Bi_2_I_9_, it was observed that the first absorption peak in dimeric Cs_3_Bi_2_I_9_ appeared at 492 nm, while a similar transition in layered Cs_3_Bi_2_I_9_ appeared to be red-shifted to 550 nm (Figure 3b). Layered Cs_3_Bi_2_I_9_ exhibits distinctive peaks that differ from dimeric Cs_3_Bi_2_I_9_, although the powder X-ray diffraction (XRD) patterns do not entirely exclude the presence of dimeric structures in the layered phase (Figure 3c). Additionally, band structure calculations using plane-wave DFT revealed that the band gap of the layered structure is lower than that of the dimeric structure. This observation is attributed to the lower conduction band minimum (CBM) in the layered structure compared to that in the dimer structure, resulting in a reduction in the bandgap. The crystal structure of Bi-PeNCs can be influenced by the heating-up process, which is commonly employed for the synthesis of colloidal inorganic quantum dots (QDs). This is because it induces the nucleation, growth, and crystallization of inorganic QDs at high temperatures. The heating-up synthesis method provides relative safety owing to the absence of injection at high temperatures and the controllability of the crystal size by varying the reaction temperature and time, making it potentially advantageous for large-scale production [82,83,84,85,86]. Building on this approach, heating-up synthesis without injection can be used to synthesize cesium bismuth bromide NCs [87]. A mixture of CsBr, BiBr_3_, OA, and OAm was stirred in ODE under vacuum, followed by heat treatment, resulting in the synthesis of Cs_3_BiBr_6_ or Cs_3_Bi_2_Br_9_ NCs. The structure of cesium bismuth bromide NCs can transform from Cs_3_BiBr_6_ to Cs_3_Bi_2_Br_9_, depending on the temperature and amount of OA. The PL and PL excitation (PLE) spectra of Cs_3_BiBr_6_ and Cs_3_Bi_2_Br_9_ are shown in Figure 3d and 3e, respectively. The emission peak of Cs_3_BiBr_6_ is observed at 435 nm, whereas that of Cs_3_Bi_2_Br_9_ is red-shifted to 461 nm. The absorption spectra corresponded to a bandgap of 3.05 and 2.67 eV, respectively.

Metal-cation doping is an efficient approach for enhancing the optoelectronic properties of PeNCs. Liu et al. synthesized Mn^2+^-doped Cs_3_Bi_2_I_9_ NCs to improve these properties [88]. In this method, MnI_2_ was added to the Bi-oleate solution, and the addition of Mn^2+^ ions suppressed and eliminated the formation of CsI impurities arising from the decomposition of the intermediate Cs_3_BiI_6_ species during synthesis. Figure 3f shows the absorption and PL spectra as a function of the Mn^2+^ doping concentration. Compared with the absorption spectrum of the undoped Cs_3_Bi_2_I_9_ NCs, that of Mn^2+^-doped Cs_3_Bi_2_I_9_ was red-shifted, accompanied by double peaks at 530–540 and 610–620 nm. This can be attributed to emission from a spin-forbidden ^4^T_1_-^6^A_1_ Mn d-d transition resulting from the transfer of exciton energy from the host Cs_3_Bi_2_I_9_ to the Mn^2+^ dopants, as commonly observed in Mn^2+^-doped LHP NCs [89,90,91]. Additionally, with increasing Mn^2+^ concentration from 1 to 5%, the intensity of the PL spectrum increased with a small spectral blue shift from 620 to 610 nm and an improved PLQY from 0.54% (undoped) to 1.57% (5% doped). Figure 3g illustrates the emission mechanism of the Mn^2+^-doped Cs_3_Bi_2_I_9_ NCs. When the Mn^2+^-doped Cs_3_Bi_2_I_9_ NCs were excited at ~510 nm, energy was transferred from the Cs_3_Bi_2_I_9_ host to the Mn^2+^ dopants, resulting in emission from the Mn^2+^. Exciton relaxation occurs through three pathways: (i) recombination within the host material assisted by phonons; (ii) non-radiative recombination involving trap sites; and (iii) radiative emission through the Mn *d*-*d* transition (^4^T_1_-^6^A_1_), facilitated by energy transfer to Mn^2+^. Through these exciton relaxation pathways, the Mn^2+^-doped Cs_3_Bi_2_I_9_ NCs exhibited two emission peaks attributed to radiative recombination within the Cs_3_Bi_2_I_9_ host and Mn^2+^ dopants, and an increase in radiative recombination leads to an enhanced PLQY.

Despite significant progress in the field of Bi-PeNCs, their PLQY remains generally low [69,70,71,72,75,87,92]. This is primarily attributed to residual surface states, strong photon-phonon coupling, and dangling bonds present on the PeNC surfaces [93,94]. To address this issue, the synthesis of MA_3_Bi_2_(Cl, Br)_9_ NCs was proposed through a collaborative ligand-assisted reprecipitation (Co-LARP) method, which leads to an improvement in the PLQY through surface passivation [95]. The added Cl anions serve as ligands on the surface of the PeNCs, reducing surface defects and inducing a blue-shifted emission spectrum. Notably, when the Cl/(Cl + Br) ratio reached 33%, the MA_3_Bi_2_(Cl, Br)_9_ NCs exhibited a maximum PLQY of 54.1%, and the PL intensity remained at 88% after 12 h under 365 nm UV irradiation.

Bi-PeNCs exhibit various crystal structures depending on their constituent elements. Their optical, photophysical, and electronic properties can be altered using different synthesis methods and doping strategies. Various approaches offer the potential for advancement by tuning and enhancing their optoelectronic characteristics. Although there are no reported cases of Bi-PeNC-based LEDs and their optical properties are inferior to those of LHP NCs, the low cost, non-toxicity, and high stability of Bi suggest that Bi-PeNCs are promising candidates for Pb replacement.

### 2.3. Sb-Based Perovskite Nanocrystals

**Figure 4:**
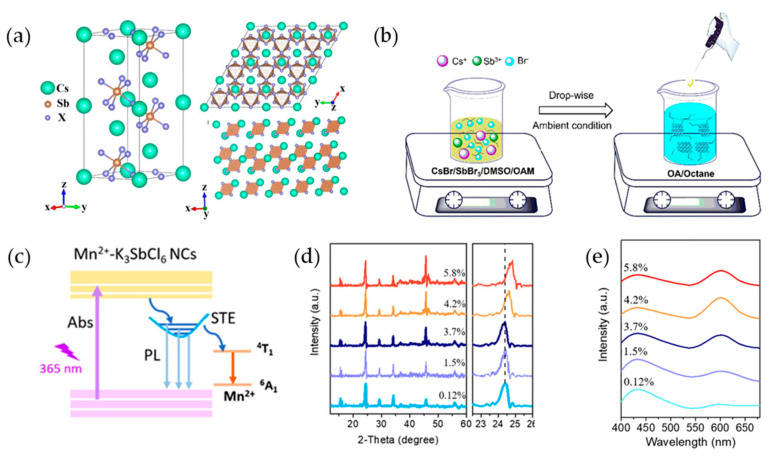
(**a**) Crystal structure of Cs_3_Sb_2_X_9_ (X = Cl, Br, and I), with a specific view of its *xy* plane illustrating the spatial distribution of Cs, Sb, and X atoms within the crystal lattice and layered form; (**a**) reprinted with permission from Ref. [96]. Copyright 2022, MDPI. (**b**) Schematic image of the Cs_3_Sb_2_Br_9_ NC precursor reaction system for the ligand-assisted reprecipitation (LARP) technique; (**b**) reprinted with permission from Ref. [98]. Copyright 2017, American Chemical Society. (**c**) The PL mechanism of Mn^2+^-doped K_3_SbCl_6_ NCs; (**d**) XRD patterns of as-prepared Mn^2+^-doped K_3_SbCl_6_ NCs at different doping concentrations; (**e**) PL spectra of Mn^2+^-doped K_3_SbCl_6_ NCs at different doping concentrations under excitation at 365 nm; (**c**–**e**) Reprinted with permission from Ref. [99]. Copyright 2020, Elsevier.

Cs_3_Sb_2_X_9_ (X = Cl, Br, and I), which has a trigonal crystal structure with space group P$\bar{3}$m1 (no. 164), consists of two [Sb_2_X_9_]^3−^ polyhedra within a single unit [96]. In the crystal structure of Cs_3_Sb_2_X_9_ shown in Figure 4a, two Sb^3+^ ions are located on the body diagonal of the unit cell, whereas the eight top corners are occupied by Cs^+^ ions. The connected [Sb_2_X_9_]^3−^ polyhedra form a bilayer structure that is stacked together. Cs_3_Sb_2_X_9_ has a layered structure and exhibits triangular symmetry.

A simple reprecipitation method did not yield Sb-PeNCs without the assistance of long-chain ligands or excessive ligand usage. Cs_3_Sb_2_Br_9_ NCs can be synthesized using the ligand-assisted reprecipitation (LARP) method. This enables room-temperature PeNC synthesis without traditional heating, an inert gas atmosphere, or injection conditions [97]. LARP involves the direct addition of a reaction precursor solution to a vigorously stirred, poor solvent to rapidly achieve a supersaturated state (Figure 4b). To initiate the LARP synthesis process, a clear precursor solution was prepared by dissolving a mixture of SbBr_3_, CsBr, and OAm in a good solvent such as N,N-dimethylformamide (DMF) or dimethylsulfoxide (DMSO). Subsequently, a fixed quantity of the prepared precursor solution was carefully dropped into a vigorously stirred solution of octane and OA, which serves as a poor solvent for the precursors. Supersaturation induces the immediate and efficient recrystallization of Cs_3_Sb_2_Br_9_ NCs, leading to their successful synthesis. By utilizing a mixture of octane and OA as ligands, Cs_3_Sb_2_Br_9_ NCs with PLQYs of ~20% were synthesized. Moreover, by further optimizing the synthesis conditions of a precursor concentration of 0.033 mM, an octane/OA ratio of 10:1, and a room-temperature reaction, monodispersed Cs_3_Sb_2_Br_9_ NCs were obtained with a high PLQY of 46% and a uniform size of 3.07 nm [98].

This doping strategy was proven to be highly effective in enhancing the optical properties of Sb-PeNCs synthesized by the hot injection method. Pristine K_3_SbCl_6_ NCs displayed a broad PL spectrum associated with self-trapped excitons (STE) and achieved a moderate PLQY of 22.3% [99]. However, through the incorporation of Mn^2+^ ions as dopants, the Mn^2+^-doped K_3_SbCl_6_ NCs exhibited a remarkable PLQY improvement to 37.2%, attaining white light emission. Furthermore, Mn^2+^-doped K_3_SbCl_6_ NCs introduce additional red emission associated with the intrinsic transition (^4^T_1_−^6^A_1_) of Mn^2+^ ions [100,101]. A primary dynamic process for the Mn^2+^-doped K_3_SbCl_6_ NCs was proposed (Figure 4c). Initially, upon excitation, the excited charge carriers cause rapid elastic distortions, leading to the formation of STE states owing to strong electron-phonon interactions within the K_3_SbCl_6_ host. Simultaneously, an energy transfer process occurs between the Mn^2+^ dopant ions and the surrounding STE states, resulting in the excitation of the Mn^2+^ dopants. 

This excitation leads to an efficient red emission spectrum through the intrinsic transitions (^4^T_1_−^6^A_1_) of the Mn^2+^ ions, contributing to an observed additional red emission in the Mn^2+^-doped K_3_SbCl_6_ NCs. The interplay of these dynamic processes not only enhances the overall PLQY but also induces the versatile luminescent properties of Mn^2+^-doped K_3_SbCl_6_ NCs, offering possibilities for optoelectronic applications. The energy transfer process was further validated using excitation spectra obtained by selectively monitoring the energy transfer from the STE at 440 nm and the intrinsic transition of Mn^2+^ ions at 600 nm. Both emission spectra exhibited similar broad excitation bands, confirming the occurrence of energy transfer between these two entities. The emission decay was significantly faster when the STE was monitored at 440 nm with increasing concentrations of Mn^2+^. This indicates that the excited energy level (^4^T_1_−^6^A_1_) of the Mn^2+^ ions primarily originates from the energy transferred from the STE of the K_3_SbCl_6_ NCs host. The faster emission decay suggests an efficient energy transfer from the perovskite host to the Mn^2+^ ions, confirming the occurrence of energy transfer between them. By controlling the doping concentration of Mn^2+^ ions, it was possible to achieve white-light emission with a PLQY of 37.2%. XRD analysis of the 4.2% Mn^2+^-doped K_3_SbCl_6_ NCs revealed that the diffraction peaks at approximately 15.1°, 24.4°, 29.3°, 34.0°, and 46.0° remained similar to those of the undoped K_3_SbCl_6_ NCs (Figure 4d). These diffraction peaks are associated with the zero-dimensional (0D) monoclinic K_3_Sb_2_Cl_6_ perovskite structure (PDF#24-0833). Interestingly, the diffraction peaks of the Mn^2+^-doped K_3_SbCl_6_ NCs showed minimal changes at Mn^2+^ ion doping concentrations of 0.12%, 1.5%, and 3.7%, and a clear red shift in the diffraction peaks was observed when the Mn^2+^ ion doping concentration exceeded 4.2%. This observation provides further evidence that Mn^2+^ ions were successfully incorporated into the lattice of the 0D K_3_SbCl_6_ host. Furthermore, the relative intensity of the STE emission, compared to the intrinsic transition of Mn^2+^ ions, decreased progressively as the Mn^2+^ ion doping concentration increased from 0.12% to 4.2% (Figure 4e). This decrease signifies the occurrence of energy transfer from the K_3_SbCl_6_ host to Mn^2+^.

Sb-PeNCs are environmentally friendly B-site substitutes and are capable of forming an A_3_B(III)_2_X_9_ structure by the heterovalent replacement of Pb cations. One of the advantages of this structure is the feasibility of low-temperature synthesis and greater thermodynamic stability compared with the 2D perovskite phase. However, the lack of a suitable synthesis method for producing high-quality Sb-PeNCs remains a limitation, leading to poor morphology and hindering their application. A doping strategy shows great potential for solid-state lighting applications but may require different approaches for high-purity displays.

### 2.4. Cu-Based Perovskite Nanocrystals

Transition metals such as Cu have gained considerable attention because of their low toxicity, abundance, excellent stability, and high PLQYs compared to other transition metal-based MHPs [102,103,104]. The small ionic radius of Cu, measuring 0.77 nm for Cu^+^ and 0.73 nm for Cu^2+^, prevents it from conforming to a tolerance factor of 1 and maintaining a 3D structure. Therefore, Cu mainly has electronic 0D or 1D structures, which have special optical properties owing to their large exciton binding energies, the A_x_Cu_y_X_x+y_ structure in monovalent form, and the A_x_Cu_y_X_x+2y_ structure in divalent form [102,105].

In the synthesis of Cu(II)-PeNCs, such as Cs_2_CuX_4_, Cs_2_CuI_4_ could not be obtained because pure Cu(II) diiodide is unstable and decomposes easily into iodine and Cu(I) iodide. Cs_2_CuCl_4_ exhibited a broad PL spectral range of 400–650 nm, with a PLQY of 51.8%. Therefore, extensive research has focused on Cu (I)-PeNCs owing to the instability and broad emission spectra of Cu(II)-PeNCs [106].

**Figure 5:**
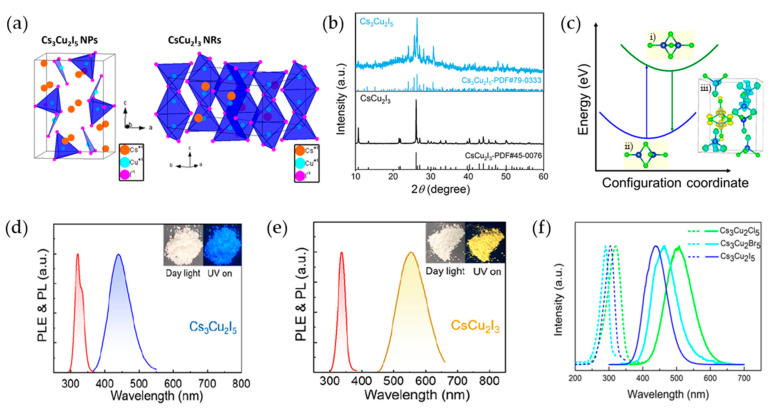
(**a**) Crystal structures of 0D Cs_3_Cu_2_I_5_ and 1D CsCu_2_I_3_; (**a**) Reprinted with permission from Ref. [107]. Copyright 2019, American Chemical Society. (**b**) XRD patterns of Cs_3_Cu_2_I_5_ and CsCu_2_I_3_; (**b**) reprinted with permission from Ref. [108]. Copyright 2022, American Chemical Society. (**c**) Schematic illustration of the principle of STE. (i) A self-trapped state; (ii) a ground state; (iii) charge density for conduction and valence bands; (**d**) PL and PLE spectra of Cs_3_Cu_2_I_5_; (**e**) PL and PLE spectra of CsCu_2_I_3_; (**d**,**e**) Reprinted with permission from Ref. [117]. Copyright 2021, American Chemical Society (**f**) PLE (Cl = 320 nm, Br = 290 nm, I = 305 nm) and PL (Cl = 515 nm, Br = 461 nm, I = 445 nm) spectra of Cs_3_Cu_2_X_5_ at room temperature; (**c**,**f**) Reprinted with permission from Ref. [111]. Copyright 2020, American Chemical Society.

Cu(I)-PeNCs exhibit structural variations depending on the A-site. When Cs is located at the A-site of Cu-PeNC, Cs_3_Cu_2_X_5_ and CsCu_2_X_3_ structures are observed (Figure 5a) [107]. The space groups of the structures were identified from their XRD patterns (Figure 5b) [108]. Cs_3_Cu_2_I_5_ belongs to the Pnma orthorhombic space group, and its crystal structure consists of [Cu_2_I_5_]^3−^ cluster units composed of tetrahedral [CuI_4_] and trigonal planar [CuI_3_] units that are separated from the Cs^+^ ions. This arrangement gives rise to an electronic 0D structure [109]. CsCu_2_I_3_ belongs to the Cmcm space group of orthorhombic crystal systems and exhibits a 1D chain-like structure. This structure is formed by tetrahedral [CuI_4_]^3−^ units that share a common edge, connecting them in a linear structure. By contrast, Cu(I)-PeNCs with Rb or K at the A-site follow the A_2_CuX_3_ structure, which belongs to the Pnma space group. They exhibit a 1D shape where tetrahedral [CuX_4_]^3−^ units share only the common vertex of the halides, resulting in a chain-like structure [110]. Overall, Cu(I)-PeNCs exhibit diverse crystal structures, including 0D and 1D forms, depending on the specific combination of the A-site and X-site compositions.

In the excited state, Cu-PeNCs show reorganization owing to the Jahn–Teller distortion that forms STE energy states and induces a Stokes shift, representing the difference between the absorption and emission spectra. In the case of Cs_3_Cu_2_X_5_, the absorption of photon energy converts Cu(I) 3d^10^ to Cu(II) 3d^9^, which changes the electronic configuration and induces a Jahn–Teller distortion (Figure 5c) [111]. Similarly, the conduction and valence bands of CsCu_2_I_3_ are also dispersed along the direction parallel to the tetrahedral [Cu_2_I_6_]^4−^ chain, but when it is excited, the electrons are localized due to strong Coulombic coupling and large structural distortion of the [Cu_2_I_6_]^4−^ tetrahedron, and the structure is reorganized [112,113,114]. When the lattice distortion energy is high, charges are trapped because the trapped state is more stable than the strained state. Therefore, the Jahn–Teller distortion, which forms STE states, effectively confines charges to localized regions. Simultaneously, as STEs are formed, the ground state energy increases owing to the lattice strain energy. Consequently, STE emission exhibits a large Stokes shift [115,116]. The emission energy (E_em_) from the STE states can be expressed as follows [116]:

E_em_ = E_g_ − E_b_ − E_st_ − E_d_ (3)

where E_g_, E_b_, E_st_, and E_d_ represent the bandgap energy, the exciton binding energy, the self-trapping energy, and the lattice strain energy, respectively. The smaller distortion in CsCu_2_I_3_ compared to the octahedral distortion in Cs_3_Cu_2_I_5_ indicates a smaller bandgap of CsCu_2_I_3_ (Figure 5d,e) [117]. Moreover, the extended PL decay lifetime of the time-resolved photoluminescence (TrPL) in Cs_3_Cu_2_I_5_ compared to that in CsCu_2_I_3_ can be attributed to two key factors: the augmented exciton binding energy and significant lattice distortion. Cs_3_Cu_2_I_5_ exhibits an enhanced exciton binding energy of 224 meV, while CsCu_2_I_3_ has a relatively low binding energy of 123 meV. The higher exciton binding energy in Cs_3_Cu_2_I_5_ decelerates the exciton decay rate, resulting in an extended PL lifetime [118,119].

By tuning the halogen anions (X = Cl, Br, and I), the emission wavelength of Cs_3_Cu_2_X_5_ can be controlled, ranging from the green wavelength region (515 nm, Cs_3_Cu_2_Cl_5_) to the blue wavelength region (445 nm, Cs_3_Cu_2_I_5_) (Figure 5f) [111]. This spectral tunability of Cs_3_Cu_2_X_5_ is attributed to the effect of the halogen size and electronegativity on the electronic structure. During electron excitation, as the halogen radius increases and electronegativity decreases, stronger hybridization between the halogen *p*-orbital and the Cu 3*d* orbital is induced [120,121]. This enhanced hybridization leads to a decrease in the valence band maximum (VBM) of Cs_3_Cu_2_X_5_. Contrary to the general trend, this decrease in the VBM increases the band gap, leading to a blue shift in the emission wavelength. This phenomenon can be attributed to electronic interactions between the halogen and Cu atoms, which modulate the energy levels and affect the emission properties.

A hot-injection method was used to synthesize 0D Cs_3_Cu_2_X_5_ and 1D CsCu_2_X_3_ by controlling the reaction temperature [118]. In this synthesis method, nucleation was induced within a short time by injecting a Cs-oleate into a Cu-oleate containing OAm and OA ligand molecules as quickly as possible and then cooling the solution. When the temperature of Cu-oleate reached 70 °C and the Cs-oleate solution was rapidly injected, 0D Cs_3_Cu_2_I_5_ NCs were synthesized, and when injected at a high temperature of 110 °C, 1D CsCu_2_I_3_ was synthesized as the final product. The synthesized Cs_3_Cu_2_I_5_ NCs exhibited blue PL at 441 nm and a PLQY of 67%, whereas the 1D CsCu_2_I_3_ NCs exhibited yellow PL at 553 nm and a PLQY of ~5%.

Using an anti-solvent precipitation method, 0D Cs_3_Cu_2_X_5_ and 1D CsCu_2_X_3_ can also be synthesized by adjusting the concentration of the precursor solution and the amount of anti-solvent [122]. The CsI and CuI precursors were dissolved in a good solvent, such as DMF or DMSO, and then an anti-solvent such as ethanol was added to precipitate the Cu-PeNC. 1D CsCu_2_I_3_ can be obtained in a low-concentration precursor solution because Cu^+^ precipitates rapidly when an antisolvent is added to a low-concentration precursor. As the concentration of the precipitated Cu^+^ ions increases, the number of ions participating in the crystallization process increases, and 1D CsCu_2_I_3_ is synthesized. By contrast, 0D Cs_3_Cu_2_I_5_ can be obtained from a high-concentration precursor solution (0.4 M) without a significant change in the Cs:Cu ratio when an antisolvent is added. The synthesized Cs_3_Cu_2_I_5_ NCs exhibited a PL emission peak at 440 nm with a full width at half maximum (FWHM) of 73 nm and a PLQY of 67%, whereas 1D CsCu_2_I_3_ exhibited a PL emission peak at 580 nm with a broad FWHM of 125 nm and a low PLQY of 7.4%.

During the hot-injection process, metal halide doping, such as with a ZnI_2_ precursor, can lead to a surface passivation effect in Cs_3_Cu_2_X_5_ NCs [123]. Additionally, Zn cation doping resulted in a tetrahedral coordination arrangement, and Cu^+^ ions were heterosubstituted with Zn^2+^ ions. This doping enriched the excited electron density, thereby enhancing the STE emission. Moreover, the incorporation of ZnI_2_ results in enriched I anions, which can effectively reduce the number of non-radiative centers caused by the iodine vacancies. Zn-doped Cs_3_Cu_2_I_5_ NCs exhibited a blue emission peak at ~440 nm with an FWHM of 73 nm. The PLQY increased from 72.2% for Cs_3_Cu_2_I_5_ NCs to 92.8% for the Zn-doped Cs_3_Cu_2_I_5_ NCs.

Cu-PeNCs can be synthesized in both the monovalent Cu^+^ and divalent Cu^2+^ forms using hot injection and antisolvent precipitation methods. In Cu(I)-PeNCs, lattice distortion arises from the structural characteristics of the PeNCs, resulting in STEs. This phenomenon contributes to a high PLQY and a prolonged PL lifetime resulting from highly localized electrons and holes. Moreover, in contrast to the typical red shift observed during the change in halide composition from Cl to I, the observed blue shift in the emission spectrum can be attributed to structural transformations and significant exciton effects. The emission wavelength and crystal structure can be controlled by adjusting the synthesis conditions, precursor ratios, and halide composition. Additionally, defect passivation can be facilitated through metal halide doping, which enhances the optical properties of the Cu-PeNCs. However, the broad FWHM of the emission spectrum stemming from the presence of STEs remains a major challenge to be solved using a novel approach in materials engineering.

### 2.5. Double Perovskite Nanocrystals

**Figure 6:**
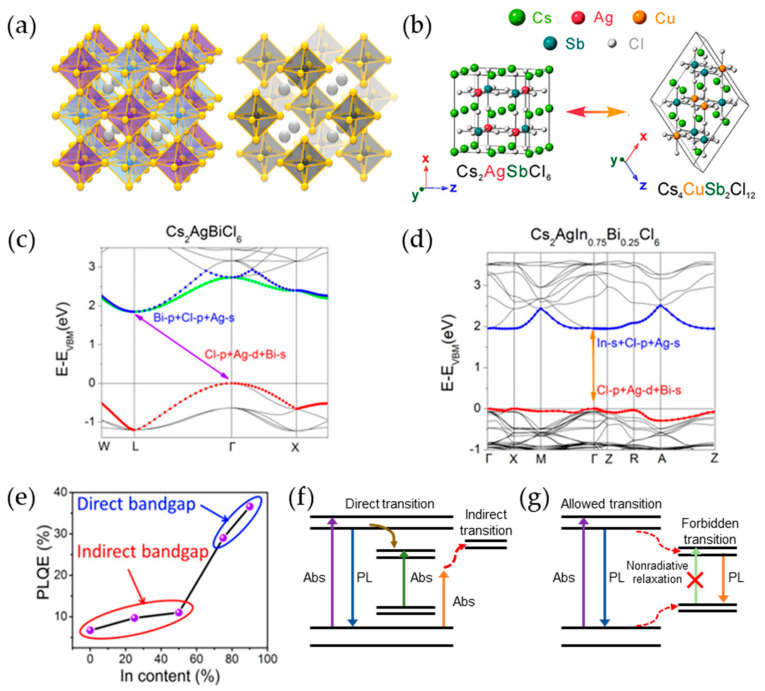
(**a**) Crystal structures of double perovskites (A_2_B(I)B(III)X_6_) and vacancy-ordered double perovskites (A_2_B(IV)X_6_); (**b**) crystal structures of Cs_4_Cu(II)Sb(III)_2_Cl_12_ and Cs_2_Ag(I)Sb(III)Cl_6_; (**b**) reprinted with permission from Ref. [124]. Copyright 2020, American Chemical Society. Band structures of (**c**) Cs_2_AgBiCl_6_ and (**d**) Cs_2_AgIn_0.75_Bi_0.25_Cl_6_; (**e**) PLQE value of Cs_2_AgIn_x_Bi_1−x_Cl_6_ (x = 0, 0.25, 0.5, 0.75, and 0.9) NCs; (**c**–**e**) reprinted with permission from Ref. [134]. Copyright 2018, American Chemical Society. Charge carrier transportation mechanism models of (**f**) indirect bandgap Cs_2_AgIn_x_Bi_1−x_Cl_6_ NCs and (**g**) direct bandgap Cs_2_AgIn_x_Bi_1−x_Cl_6_ NCs.

Pb-free double perovskites consist of two different cations occupying the B-sites of an A_2_BB’X_6_ structure, providing high flexibility in the selection of B-site cations. The double perovskite structure maintains electrical neutrality by substituting two B^2+^ ions with a B^+^ cation and a B^3+^ cation, forming an A_2_B(I)B(III)X_6_ structure (Figure 6a, left). The B-site can accommodate monovalent cations such as K^+^, Na^+^, and Ag^+^ and trivalent cations such as Bi^3+^, In^3+^, and Sb^3+^. Another structural variant is vacancy-ordered double perovskites, where two B^2+^ ions are replaced by a single tetravalent cation, such as Sn^4+^ or Ti^4+^, and B-site vacancies, forming the A_2_B(IV)X_6_ structure (Figure 6a, right). Additionally, a 3D-layered structure with one vacancy can be achieved when four B^2+^ ions are substituted by one B^2+^ ion and two B^3+^ ions, as represented by vacancy-ordered quadruple perovskites A_4_B(II)B(III)_2_X_12_. The incorporation of one Cu^2+^ ion and two Sb^3+^ ions in place of four Pb^2+^ ions generates a single vacancy, resulting in the formation of a monoclinic layered double perovskite structure such as Cs_4_CuSb_2_Cl_12_ (Figure 6b) [124]. Thus, the combination of B-site cations and vacancies results in a wide range of structural variations.

Double PeNCs have gained significant attention as promising candidates to replace Pb because of their tunable optoelectronic properties; high stability against heat, humidity, and light; and versatility in selecting B-site cations [125,126,127,128]. However, their commercial application is hindered by poor PL characteristics, which are attributed to intrinsic and surface defects, indirect bandgaps, and forbidden transitions [129,130,131,132]. To address these challenges, various strategies have been explored to improve their optoelectronic properties.

Ag^+^Bi^3+^-PeNCs exhibit low PLQYs, mainly owing to their indirect bandgap characteristics [126,129,133]. The absorption tail observed in the long-wavelength region of the absorption spectrum indicates sub-bandgap absorption [70,132]. Yang et al. introduced changes in the indirect bandgap characteristics by doping In^3+^ ions in Cs_2_AgBiCl_6_ NCs [134]. Optical analysis of the Cs_2_AgIn_x_Bi_1−x_Cl_6_ NCs revealed blue-shifted absorption peaks with increasing In^3+^ doping concentration, and sharp absorption peaks were observed at x values of 0.75 and 0.9, indicating possible direct bandgap characteristics. The intensity of the PL emission peak observed at ~400 nm increased with increasing In^3+^ doping concentration, and a second peak appeared at ~570 nm at x > 0.75.

To gain a deeper understanding of the optical mechanism and bandgap characteristics, DFT calculations were performed using the generalized gradient approximation of the Perdew–Burke–Ernzerhof functional. Figure 6c,d depict the band structures of Cs_2_AgIn_x_Bi_1−x_Cl_6_ NCs with In ratios of 0% and 75%, respectively. The band structure of Cs_2_AgBiCl_6_ NC exhibits an indirect bandgap, whereas that of Cs_2_AgIn_0.75_Bi_0.25_Cl_6_ NC shows a nearly direct bandgap. In the Cs_2_AgBiCl_6_ NC, the VBM at the Γ point has Cl-*p*, Ag-*d*, and Bi-*s* characteristics, whereas the CBM at L has Bi-*p*, Cl-*p*, and Ag-*s* characteristics. Conversely, in the Cs_2_AgIn_0.75_Bi_0.25_Cl_6_ NC, the VBM at the Γ point has Cl-*p*, Ag-*d*, and Bi-*s* characteristics, similar to those of the Cs_2_AgBiCl_6_ NC, whereas the CBM comprises In-*s*, Cl-*p*, and Ag-*s* characteristics, similar to those of the Cs_2_AgInCl_6_ NC. This indicates the possibility of direct transitions, as observed in the band structure of the Cs_2_AgIn_0.75_Bi_0.25_Cl_6_ NC. Similar to Cs_2_AgInCl_6_ NC, where a parity-forbidden transition from the VBM to the CBM occurs at the Γ point, Cs_2_AgIn_0.75_Bi_0.25_Cl_6_ NC provides a pathway for relaxation through parity-forbidden transitions, leading to emission at approximately 570 nm [135]. These results suggest that the dual PL emission peaks can be attributed to the emissions resulting from both band- and parity-forbidden transitions. The PLQYs showed a pronounced enhancement above 0.75 in the doping ratio, resulting in a PLQY of 36.6% in Cs_2_AgIn_0.75_Bi_0.25_Cl_6_ NC, but a slight increase below 0.5 in the doping ratio, resulting in a PLQY of ~10% in Cs_2_AgIn_0.5_Bi_0.5_Cl_6_ NC. This substantial improvement in the PLQY above 0.75 in the ratio originates from the transition from an indirect bandgap to a direct bandgap (Figure 6e). Band structure diagrams in Figure 6f,g depict the photoinduced charge carrier transportation mechanism and provide an intuitive understanding of the absorption and emission processes based on the indirect and direct bandgap characteristics of the double PeNCs. In the case of an indirect bandgap, both direct and indirect transitions can occur. These indirect bandgap transitions require phonon participation, resulting in a low PLQY [136,137]. However, for a direct bandgap, absorption occurs through direct transitions, and the excited carriers can undergo non-radiative relaxation processes to the forbidden states, resulting in two forms of luminescence: direct bandgap relaxation and relaxation to the forbidden states. Radiative relaxation of the band edge and forbidden states improved the PLQY of Cs_2_AgIn_x_Bi_1−x_Cl_6_ when x was greater than 0.75.

Cs_2_SnX_6_-based vacancy-ordered double perovskites utilizing Sn^4+^ have attracted significant research interest owing to their air and moisture stability, high absorption coefficients, and carrier mobility [138,139,140]. Wang et al. reported a synthesis method for Cs_2_SnI_6_ perovskites that could be adjusted from spherical NCs, nanorods, nanowires, and nanobelts to nanoplatelets by varying the reaction time during the hot-injection process [141]. However, the PLQY of the Cs_2_SnI_6_ NCs was found to be as low as 0.48%. Cs_2_SnI_6_ NCs can also be synthesized by a ligand-mediated approach using the hot-injection method, similar to conventional LHP NC synthesis [142,143,144]. By adjusting the ratio of OA to octylamine, the structure of the Cs_2_SnI_6_ NCs could be controlled between the 3D and 2D forms. When only OA was used during the synthesis, 3D Cs_2_SnI_6_ NCs were formed, whereas the combination of OA and octylamine resulted in the formation of 2D Cs_2_SnI_6_ nanoplatelets. The one-layer 2D Cs_2_SnI_6_ exhibited a PL emission peak at 643 nm, while the two-layer 2D Cs_2_SnI_6_ displayed a PL emission peak at 742 nm. The PLQYs were measured as 28% for the one-layer Cs_2_SnI_6_ and 16% for the two-layer Cs_2_SnI_6_. These results can be attributed to the quantum confinement effect of the quantum-well structure, which enhances the exciton binding energy and improves the PL characteristics [145,146].

**Figure 7:**
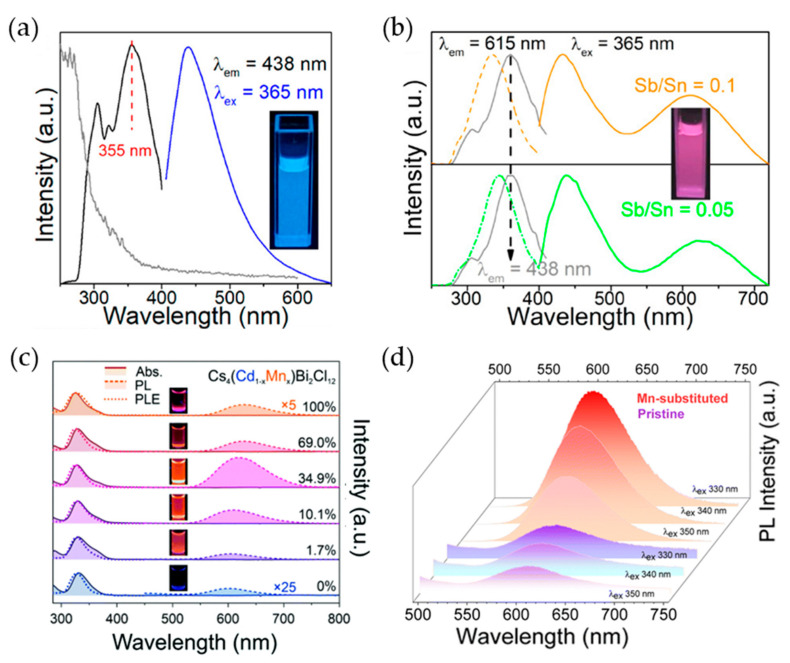
(**a**) Absorption (gray line) excitation (dashed line, emission at 438 nm) and PL (excitation at 365 nm) spectra of undoped Cs_2_SnCl_6_ NCs (inset: UV irradiation under 365 nm UV lamp); (**b**) PLE (dashed line, emission at 615 nm; gray line, emission at 438 nm) and PL (excitation at 365 nm) spectra of Sb-doped Cs_2_SnCl_6_ NCs (inset: UV irradiation of Sb/Sn = 0.1 NCs under 365 nm UV lamp); (**a**,**b**) reprinted with permission from Ref. [147]. Copyright 2019, American Chemical Society. (**c**) Absorption, PLE, and PL spectra of Cs_4_Cd_1−x_Mn_x_Cl_12_ NCs (inset: UV irradiation under UV light); (**c**) reprinted with permission from Ref. [152]. Copyright 2020, Royal Society of Chemistry. (**d**) Room temperature PL spectra of Cs_4_CdBi_2_Cl_12_ (pristine) and Cs_4_Cd_0.6_Mn_0.4_Bi_2_Cl_12_ (Mn-substituted) nanosheet (NS); (**d**) reprinted with permission from Ref. [157]. Copyright 2023, Royal Society of Chemistry.

Similar to the behavior of other PeNCs, doping of metal ions, such as Sb^3+^, in Cs_2_SnCl_6_ NCs is an effective strategy for enhancing their luminescent properties [147]. Pristine Cs_2_SnCl_6_ NCs synthesized by the hot-injection process exhibited a sharp absorption edge at 310 nm with an absorption tail at longer wavelengths (Figure 7a). Under 365 nm excitation, the pristine Cs_2_SnCl_6_ NCs showed a PL spectrum peak at 438 nm with a PLQY of 4.37%. Sb^3+^ doping of the Cs_2_SnCl_6_ NCs was achieved with Sb/Sn precursor ratios of 0.05 and 0.1, where SbCl_3_ was mixed concurrently with SnCl_2_. The Sb^3+^-doped Cs_2_SnCl_6_ NCs exhibited double PL peaks at 438 and 615 nm, and the PL intensity at 615 nm increased with increasing Sb^3+^ concentration from 0.05 to 0.1 (Figure 7b). Further investigations revealed that the 615 nm emission could be attributed to the triplet states of STE induced by Sb^3+^ doping. The emission at 615 nm originates from recombination with the ground states through transitions from the singlet states of STE with emission at ~450 nm to the triplet states of STE with emission at 615 nm, resulting in broad emission wavelengths. Through Sb^3+^ doping at a ratio of 0.1, the PLQY of Sb^3+^-doped Cs_2_SnCl_6_ was enhanced to 8.25% compared to that of pristine Cs_2_SnCl_6_ (4.37%).

Vacancy-ordered quadruple perovskite A_4_B(I)B(III)_2_X_12_ has attracted significant research interest because of its large compositional space, direct bandgap characteristics, and excellent structural stability [148,149,150,151]. It consists of [M(II)X_6_]^4−^ octahedra layers sandwiched between [M(III)X_6_]^3−^ octahedra layers, crystallizing in the R$\bar{3}$m space group symmetry perpendicular to the <111> direction of the cubic perovskite structure. However, their practical applications have been hindered by their poor luminescence properties. Therefore, research has focused on enhancing the luminescent characteristics of vacancy-ordered quadruple perovskites through metal cation doping [124,152,153,154,155,156,157]. The synthesis of Cs_4_Cd_1−x_Mn_x_Bi_2_Cl_12_ NCs was proposed using a hot-injection method with benzoyl chloride [152]. The [Mn]/([Mn] + [Cd]) ratios in the final products were quantified as 0%, 1.7%, 10.1%, 34.9%, 69.0%, and 100% using inductively coupled plasma atomic emission spectroscopy. Despite an increase in the Mn ratio, the absorption and PLE spectra showed minimal changes, whereas there was a distinct red shift in the PL emission spectra (Figure 7c). The Cs_4_Cd_1−x_Mn_x_Bi_2_Cl_12_ NCs with x = 0.349 exhibit the highest PLQY of 4.6%. However, as the value of x increased further, the PLQY decreased. In Figure 7c, the Stokes shift originated from a difference between absorption and emission peaks. The influences of Stokes shift and self-absorption in PeLEDs have been commonly explained in two ways. First, self-absorption can cause photon loss due to a difference between the numbers of internally generated and externally emitted photons that can be measured as PLQYs [158]. However, the large Stokes shift could prevent self-absorption loss because the absorption spectrum does not overlap with the emission wavelength. Therefore, reduced self-absorption resulting from the Stokes shift could enhance emission efficiencies in LEDs. Secondly, however, the Stokes shift could be observed along with the increase in Auger recombination affected by the large exciton binding energy, which can lead to increased efficiency roll-off in LEDs [159,160,161]. Therefore, further systematic analysis should be studied to clearly establish the effects of the Stokes shift on LED performance.

Similar PL characteristics were observed for Cs_4_Cd_1−x_Mn_x_Bi_2_Cl_12_ nanosheets (NS) with the same constituent elements. Bhardwaj et al. synthesized Cs_4_Cd_0.6_Mn_0.4_Bi_2_Cl_12_ and 2- to 3-layer-thick 2D NS [157]. Optical characterization of pristine Cs_4_CdBi_2_Cl_12_ and Cs_4_Cd_0.6_Mn_0.4_Bi_2_Cl_12_ NS revealed that a weak PL emission appeared at ~605 nm for the pristine Cs_4_CdBi_2_Cl_12_ NS, whereas the Cs_4_Cd_0.6_Mn_0.4_Bi_2_Cl_12_ NS showed a gradual increase in the PL emission peak intensity at ~601 nm with increasing Mn doping concentration, unaffected by different excitation wavelengths (Figure 7d). These red-shifted PL spectra due to Mn doping were attributed to an inter-Mn-distance-induced Mn–Mn coupling interaction, leading to a larger *d*–*d* splitting of the Mn^2+^ ion centers, consequently reducing the gap in the ^4^T_1_-^6^A_1_ electronic transition of Mn^2+^ ions [152,162,163]. In Cs_4_CdBi_2_Cl_12_, the emission arises from the STE state, whereas the emission in Cs_4_Cd_1−x_Mn_x_Bi_2_Cl_12_ arises from energy transfer to Mn^2+^ in the STE states [153].

Double PeNCs also have limitations in practical applications owing to issues concerning instability due to metal ion oxidation, the formation of deep trap states, and a broad FWHM of the emission spectrum. To overcome these issues, ongoing research aims to enhance the photoelectric performance of double PeNCs through various doping strategies, synthesis methods, and control of variables such as ligands [164,165,166,167,168,169,170,171,172,173,174,175,176,177,178,179,180,181,182]. The characterization of changes induced by process variables will lead to diverse applications in various optoelectronic fields.

Based on the above discussion, the optical and photophysical properties of the diverse PeNCs are summarized in Table 1. It is worth noting that certain instances of Pb-free PeNCs exhibit superior PL properties in comparison to LHP NCs. In particular, for remarkable PLQYs of Cu-based PeNCs, it could be necessary to focus on mainly developing device engineering such as device physics, light out-coupling, and charge balance as future promising eco-friendly LEDs. On the other hand, for relatively low PLQYs of Sn-, Bi-, and double PeNCs, research on further developing their intrinsic material properties might be more crucial and would be helpful to further enhance PL properties through promising strategies such as defect passivation, ligand modification, surface engineering, and improved synthesis techniques.

## 3. Pb-Free Perovskite Nanocrystal-Based LEDs

Pb-free perovskite LEDs (PeLEDs) exhibit great potential as next-generation LEDs. This is due to their nontoxicity, cost-effectiveness, solution processability, facile emission wavelength tunability, and defect tolerance. Research on colloidal PeNC-based LEDs has recently gained attention in parallel with the rapid advancement of solution-based synthesis of bulk MHPs [183,184,185,186,187,188,189]. Optimizing the dimensionality of MHPs provides another avenue for tuning their electronic and optical properties [190,191,192]. PeNCs possess distinct electrical and optical properties attributable to their large exciton binding energy, tunable NC size, and reduced dimensionality [19,193].

**Figure 8:**
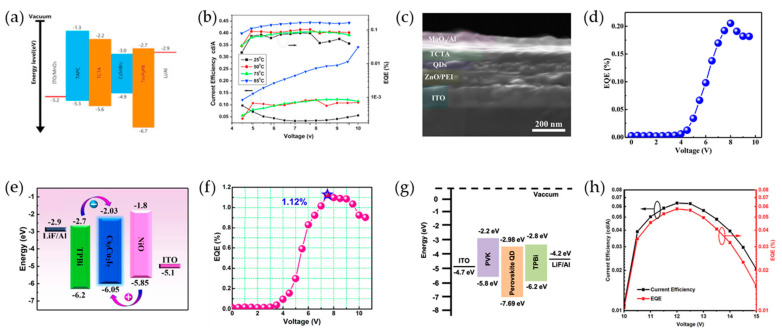
(**a**) Energy diagram of the PeLED with CsSnBr_3_ PeNCs; (**b**) current efficiency (CE) and external quantum efficiency (EQE) versus voltage curves of the CsSnBr_3_ NC-based PeLED at various in-situ annealing temperatures; (**a**,**b**) Reprinted with permission from Ref. [183]. Copyright 2020, Elsevier. (**c**) Cross-sectional scanning electron microscopy (SEM) image of ITO/ZnO nanoparticles (NP)/PEI/Cs_3_Sb_2_Br_9_ NCs/TCTA/MoO_3_/Al; (**d**) EQE-voltage curve of Cs_3_Sb_2_Br_9_ NC-based PeLED; (**c**,**d**) reprinted with permission from Ref. [186]. Copyright 2020, American Chemical Society. (**e**) Energy band of the Cs_3_Cu_2_I_5_ NC-based LED; (**f**) EQE-voltage curve of Cs_3_Cu_2_I_5_ NCs PeLED; (**e**,**f**) reprinted with permission from Ref. [187]. Copyright 2020, American Chemical Society. (**g**) Energy band diagram of Cs_2_AgIn_0.9_Bi_0.1_Cl_6_ NC-based PeLED; (**h**) current efficiency-voltage curve (black line) and EQE-Voltage curve of Cs_2_AgIn_0.9_Bi_0.1_Cl_6_ NC-based PeLED; (**g**,**h**) reprinted with permission from Ref. [188]. Copyright 2021, Wiley-VCH.

Most Sn-based MHPs are solution-processed for fabricating LEDs because of their simplicity and low-cost processability. However, the rapid crystallization of Sn-MHPs can lead to poor morphology and low film quality, which degrades the performance of LEDs [194,195,196]. To overcome the intrinsic limitations of Sn-based PeLEDs, high-vacuum vapor deposition is an alternative approach for the fabrication of Sn-PeNC films [197,198]. The fabrication of PeLEDs based on CsSnBr_3_ PeNCs can be achieved by thermal evaporation with in-situ annealing [199]. To promote the formation of CsSnBr_3_ NCs films, a dual source was used for the thermal evaporation of CsBr and SnBr_2_. Subsequently, in the post-synthetic annealing stage, the initially stacked CsBr and SnBr_2_ layers interact at the interface, resulting in the formation of a CsSnBr_3_ layer through interdiffusion of the constituent elements facilitated by thermal annealing [183]. When testing various post-synthetic annealing temperatures, the CsSnBr_3_ films annealed at 85 °C showed a significant enhancement in crystallinity. This is evident from the increased diffraction intensity of the (100) and (200) planes and a reduced FWHM. The improved crystallinity can be attributed to an enhanced reaction between the CsBr and SnBr_2_ precursors, which occurs as a result of interdiffusion. In addition, the CsSnBr_3_ NCs obtained by the annealing-temperature process exhibited an average crystal size of 39.9 nm. The devices were fabricated using the structure of ITO/MoO_3_/4,4′-cyclohexylidenebis[N,N-bis(p-tolyl)aniline]/tris(4-carbazoyl-9-ylphenyl)amine (TCTA)/CsSnBr_3_ NCs/1,3,5-tri(m-pyrid-3-yl-phenyl)benzene (TmPyPB)/LiF/Al (Figure 8a). The CsSnBr_3_ NC-based LED demonstrated a turn-on voltage of 5.5 V (at 1 cd/m^2^) and a maximum luminance of 43 cd/m^2^ at an applied voltage of 10 V. Notably, it achieved a high current efficiency (CE) of 0.34 cd/A and an external quantum efficiency (EQE) of 0.16% (Figure 8b). At an applied voltage from 4.5 to 10 V, the CsSnBr_3_ NC-based LED exhibited consistent electroluminescence (EL) emission peaks at approximately 675 nm, indicating exceptional EL stability.

Bi-based PeNC LEDs have not been reported yet. This may be caused by the low PLQY resulting from surface defects, which are due to the intrinsic self-trapping-induced phonon-mediated nonradiative process [93,95]. It could also be attributed to strong photon-phonon coupling and dangling bonds present on the PeNC surfaces [70,93,94]. As there are no reports on Bi-based PeNCs LED yet, further research is necessary to improve PLQY and engineering for LED applications.

Sb^3+^, possessing an electronegativity similar to that of Pb^2+^ ions, demonstrates exceptional structural stability and outstanding luminescent properties, making it a promising candidate for incorporation as an emitting layer (EML) in LEDs. Stable violet-emitting Cs_3_Sb_2_Br_9_ NCs, synthesized using the LARP method, exhibited a high PLQY of 51.2% [186]. Moreover, the Cs_3_Sb_2_Br_9_ NCs exhibited a moderate PL stability change of ~17.5% after 73 h of continuous UV irradiation (365 nm, 30 W). Furthermore, the PL intensity of the Cs_3_Sb_2_Br_9_ NCs was maintained at approximately 80% of the initial emission intensity when 0.5 mL of deionized water was added to the Cs_3_Sb_2_Br_9_ NC solution for 45 h, while the CsPbBr_3_ NCs experienced rapid fluorescence quenching, retaining only ~9% of the initial emission intensity for only 4 h. Moreover, the fabricated PeLED with the structure ITO/ZnO/PEI/Cs_3_Sb_2_Br_9_ NCs/TCTA/MoO_3_/Al exhibited a maximum EQE of ~0.206% and luminance of 29.6 cd/m^2^ at an operating voltage of 8.0 V (Figure 8c,d). The EL intensity peak was maintained at a wavelength of 408 nm after 6 h of device operation, and the PeLED demonstrated excellent operational EL stability, with only ~10% emission decay.

Cu-based PeLEDs are attracting attention as blue-light emitters owing to their nontoxicity, high stability, and excellent optoelectronic properties. Wang et al. reported a Cu(I)-based PeLED with the structure ITO/NiO/Cs_3_Cu_2_I_5_ NCs/2,2′,2″-(1,3,5-Benzinetriyl)-tris(1-phenyl-1-H-benzimidazole) (TPBi)/LiF/Al [187]. Cs_3_Cu_2_I_5_ NCs synthesized by the hot-injection method were employed as an EML fabricated by the spin-coating process. The energy band diagram of ITO/NiO/Cs_3_Cu_2_I_5_ NCs/TPBi/LiF/Al was obtained using ultraviolet photoelectron spectroscopy (UPS) (Figure 8e). The device structure illustrates that p-NiO possesses a relatively high ionization potential energy, making it a suitable hole-donating and electron-blocking layer because of its compatible electron affinity with the Cs_3_Cu_2_I_5_ NCs [200]. Similarly, TPBi served the dual function of electron donation and a hole-blocking layer owing to its low, lowest unoccupied molecular orbital and highest occupied molecular orbital energy levels. Electrons were injected from the upper TPBi layer into the Cs_3_Cu_2_I_5_ NC EML, facilitating EL emission through the recombination of confined electrons and holes.

Cs_3_Cu_2_I_5_ NC-LED exhibited a Commission International de I’Eclairage (CIE) coordinate of (0.16, 0.07) at 7.0 V, in compliance with the blue coordinate of the National Television Standards Committee (NTSC) standard, reaching ~99% of the NTSC standard and contributing to a wide color gamut for Pb-free PeLEDs. Additionally, the maximum EQE of the Cs_3_Cu_2_I_5_ NC-LEDs was ~1.12% (Figure 8f), with high reproducibility and a small relative deviation of 15.7%. Furthermore, Cs_3_Cu_2_I_5_ NC-LED demonstrated continuous, relatively stable device operation for 170 h at an applied voltage of 6.7 V while maintaining an EL wavelength of 445 nm. These results indicate the excellent device performance and operational stability of Cs_3_Cu_2_I_5_ NC-LEDs.

Double PeNCs face challenges in LED implementation, owing to their intrinsic and surface defects, indirect bandgaps, and suppressed emission characteristics [129,130,131,132]. However, attempts have been made to improve the optical properties of LED implementation through bandgap modification by metal cation doping and changes in carrier dynamics. Zhang et al. first developed electrically excited white LEDs based on Cs_2_AgIn_0.9_Bi_0.1_Cl_6_ NCs without a phosphor [188]. Cs_2_AgIn_0.9_Bi_0.1_Cl_6_ NCs were synthesized using a hot-injection method with TMS-Cl injection. The Cs_2_AgIn_0.9_Bi_0.1_Cl_6_ NCs exhibited two absorption peaks at 333 and 367 nm, a broad dual-color emission from STE emission, and a PLQY of 31.4%. The energy band structure of Cs_2_AgIn_0.9_Bi_0.1_Cl_6_ NCs was determined by UPS measurements, revealing a CBM of −7.69 eV and a VBM of −2.98 eV obtained by a Tauc plot. DFT calculations demonstrated a direct bandgap for Cs_2_AgIn_0.9_Bi_0.1_Cl_6_, and band symmetry analysis indicated that the parity-forbidden transition is broken at the deep-energy level of the conduction band because of the contribution of the Bi atom [135]. A device was fabricated with the structure ITO/Poly(9-vinylcarbazole) (PVK)/Cs_2_AgIn_0.9_Bi_0.1_Cl_6_ NCs/TPBi/LiF/Al (Figure 8g). The EL emission spectrum remained unchanged with increasing applied voltage, and the turn-on voltage and maximum luminance of 34.7 cd/m^2^ were measured at 10 V. The white Cs_2_AgIn_0.9_Bi_0.1_Cl_6_ NCs LED exhibited a maximum CE and EQE of 0.058 cd/A and 0.064%, respectively, and emitted white light with CIE chromaticity coordinates (0.32, 0.32) (Figure 8h).

A PeLED utilizing 2D Cs_2_AgIn_x_Bi_1−x_Cl_6_ alloyed double perovskite nanoplatelets (NPLs) was also fabricated [189]. A PeLED with the structure ITO/PEDOT: PSS/poly[(9,9-dioctylfluorenyl-2,7-diyl)-co-(4,40-(N-(4-sec-butylphenyl)) diphenylamine)] (TFB)/PVK:NPLs/ZnO/Al was fabricated. To enhance the dispersity of the NPLs and prevent electron leakage into the TFB layer, PVK was employed as a buffer component in the emissive layer, whereas ethanol-based ZnO was spin-coated as the solution-processed ETL. The Cs_2_AgIn_0.9_Bi_0.1_Cl_6_ NPL-based PeLED exhibited a single EL peak at 557 nm with a maximum luminance of 58 cd/m^2^ and an EQE of 0.01%.

## 4. Conclusions

In this review, we presented a comprehensive overview of recent advances in Pb-free PeNCs and their LED applications. PeNCs exhibit size-tunable properties, high PL efficiency, stability, and large exciton binding energy, making them promising candidates for various optoelectronic applications. However, the toxicity of Pb in LHP NCs has limited their commercialization and driven research on Pb-free MHPs.

We systematically reviewed the crystal structure, synthesis methods, efficient strategies for optoelectronic performance, and LED applications of Pb-free PeNCs based on promising B-site metal candidates as substitutes for Pb. We also discussed the synthetic strategies employed in the development of Pb-free NCs involving the optimization of crystal structures that tune their optoelectronic properties, including the PL characteristics and charge-carrier dynamics mechanism. However, the lower performance and stability, along with a broad emission wavelength of Pb-free PeNCs in comparison to LHP NCs, remain a challenge, and LED applications of Pb-free PeNCs are currently limited. These issues present difficulties for their commercialization.

**Figure 9:**
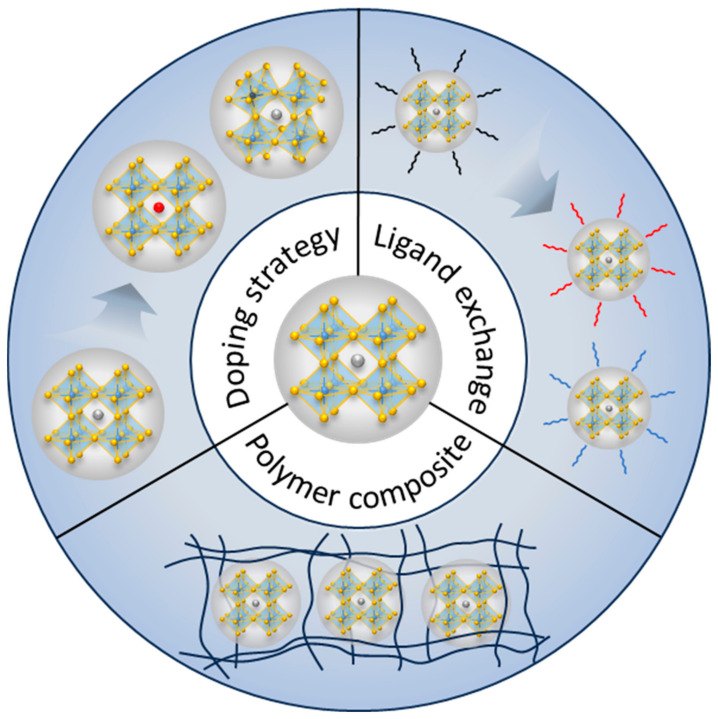
Illustration of techniques for enhancing the material properties of PeNCs.

Therefore, further investigations are required to explore strategies aimed at improving the material stability, optoelectronic properties, and device performance of Pb-free PeNCs. Active research and enhancements need to be undertaken with respect to electrical transport capabilities, optical properties, and stability to contribute to the realization and performance enhancement of LEDs. As mentioned earlier, research endeavors aimed at improving these properties necessitate the further development and utilization of techniques such as doping strategy, ligand exchange, and polymer composites (Figure 9). The directions of this material research could be a promising starting point for improving the performance of Pb-free PeNC-based LEDs. Furthermore, as research progresses on performance, stability improvement, and narrow emission spectra, Pb-free PeNCs will demonstrate the huge potential for LED development, offering prospects for a more sustainable and eco-friendly future in the field of display-related industries.

## Figures and Tables

**Table 1:** Optical and photophysical properties of PeNCs.

Perovskite	Absorption Peak (nm)	PL Peak (nm)	Stokes Shift (nm)	PLQY (%)	PL Lifetime (ns)	Reference
CsPbCl_3_	-	408	-	65	7.63	[24]
CsPbBr_3_	-	512	-	92	12.52	[24]
CsPbI_3_	-	691	-	58	20.99	[24]
FAPbBr_3_	-	545	28	-	18.7	[25]
CsPb(Br/I)_3_ NC film	-	~650	-	15	26.2	[32]
CsSnBr_3_ nanocages	655	685	30	2.1	6.52	[59]
PFOA-CsSnBr_3_ nanocages	-	683	-	1.8	0.25	[59]
OA/OAm-CsSnCl_3_	~300	~440	~140	-	7.33	[61]
Gelatin-CsSnCl_3_	303	442	139	-	8.84	[61]
Cs_3_Bi_2_Br_9_	439	460	21	4.5	~2.7	[70]
Cl-MA_3_Bi_2_Br_9_	388	422	34	54.1	2.17	[95]
Cs_3_Sb_2_Br_9_	375	410	35	46	4.285	[97]
K_3_SbCl_6_	~328	440	120	22.3	535.2	[99]
K_3_SbCl_6_:Mn^2+^	318~320	600	280	37.2	3.2 × 10^6^	[99]
CsCu_2_I_3_	321	561	240	11	0.1 × 10^3^	[107]
Cs_3_Cu_2_I_5_	300	440	158	72.2	2.8 × 10^3^	[122]
Cs_3_Cu_2_I_5_:InI_3_	295	440	145	96.6	1491	[102]
Cs_2_CuCl_4_	~300	388	88	51.8	-	[106]
Cs_2_CuBr_4_	360	393	33	37.5	-	[106]
Cs_2_AgBiBr_6_	500	625	125	-	7.5	[128]
Cs_2_AgBiCl_6_	~360	395	~35	6.7	-	[132]
Cs_2_SnCl_6_	317	438	121	4.37	11	[147]
Cs_4_CdBi_2_Cl_12_	331	~602	~271	<0.1	-	[152]

## Data Availability

Not applicable.
